# Radiomics-based prediction of two-year clinical outcome in locally advanced cervical cancer patients undergoing neoadjuvant chemoradiotherapy

**DOI:** 10.1007/s11547-022-01482-9

**Published:** 2022-03-24

**Authors:** Rosa Autorino, Benedetta Gui, Giulia Panza, Luca Boldrini, Davide Cusumano, Luca Russo, Alessia Nardangeli, Salvatore Persiani, Maura Campitelli, Gabriella Ferrandina, Gabriella Macchia, Vincenzo Valentini, Maria Antonietta Gambacorta, Riccardo Manfredi

**Affiliations:** 1 Fondazione Policlinico Universitario “Agostino Gemelli” IRCCS, 00168 Roma, Italy; 2 Università Cattolica del Sacro Cuore, Largo Francesco Vito, 1, 00168 Roma, Italy; 3 Mater Olbia Hospital, 07026 Olbia, SS Italy; 4 Gemelli Molise Hospital, Università Cattolica del Sacro Cuore, Campobasso, Italy

**Keywords:** Cervix uteri, Neoadjuvant chemotherapy, Magnetic resonance, Radiomics, Predictive medicine, Personalized medicine

## Abstract

**Purpose:**

The aim of this study is to determine if radiomics features extracted from staging magnetic resonance (MR) images could predict 2-year long-term clinical outcome in patients with locally advanced cervical cancer (LACC) after neoadjuvant chemoradiotherapy (NACRT).

**Materials and methods:**

We retrospectively enrolled patients with LACC diagnosis who underwent NACRT followed by radical surgery in two different institutions.

Radiomics features were extracted from pre-treatment 1.5 T T2w MR images.

The predictive performance of each feature was quantified in terms of Wilcoxon–Mann–Whitney test. Among the significant features, Pearson correlation coefficient (PCC) was calculated to quantify the correlation among the different predictors. A logistic regression model was calculated considering the two most significant features at the univariate analysis showing the lowest PCC value.

The predictive performance of the model created was quantified out using the area under the receiver operating characteristic curve (AUC).

**Results:**

A total of 175 patients were retrospectively enrolled (142 for the training cohort and 33 for the validation one).

1896 radiomic feature were extracted, 91 of which showed significance (*p* < 0.05) at the univariate analysis. The radiomic model showing the highest predictive value combined the features calculated starting from the gray level co-occurrence-based features. This model achieved an AUC of 0.73 in the training set and 0.91 in the validation set.

**Conclusions:**

The proposed radiomic model showed promising performances in predicting 2-year overall survival before NACRT. Nevertheless, the observed results should be tested in larger studies with consistent external validation cohorts, to confirm their potential clinical use.

**Supplementary Information:**

The online version contains supplementary material available at 10.1007/s11547-022-01482-9.

## Introduction

Cervical cancer (CC) is the fourth most frequent cancer and the fourth leading cause of cancer death in women worldwide [1].

The recommended standard treatment of patients with locally advanced cervical cancer (LACC) is currently represented by concomitant chemoradiotherapy (CRT), administered with weekly cisplatin followed by intrauterine brachytherapy [2, 3].

Despite the recent improvements related to these therapeutic strategies, the long-term overall survival rate still ranges about 57–67%, urging novel therapeutic strategies [4].

Local recurrence and distant metastasis remain the main causes of therapeutic failure in CC, proving that the microscopic residual disease, often undetectable with current diagnostic restaging strategies, represents the major risk factor for treatment failure observed after CRT.

In patients with limited response to CRT, neoadjuvant chemoradiotherapy (NACRT) followed by radical hysterectomy seems to be a valuable option [5–7].

Several experiences demonstrated that surgery has the potential to remove radio- and chemo-resistant neoplastic foci, improving local control and possibly overall survival. [8–10].

In addition, surgery represents a valid alternative to utero-vaginal brachytherapy, where brachytherapy equipment and specialists are or limited.

Magnetic resonance imaging (MRI) is currently considered the standard technique for CC local staging and prognosis evaluation before treatment, where T2-W and diffusion-weighted imaging (DWI) represent the mainstay of diagnostic sequences [11, 12].

A recent pilot study underling the efficacy of MRI in the assessment of treatment response after neoadjuvant chemotherapy plus cold knife conization, in patient affected by early CC (FIGO stage IB2- IIA1) [13].

Radiomics is a translational field of research consisting of mathematical-statistical procedures for extracting data from standard radiological images, resulting in quantitative features that describe tumor heterogeneity and other intrinsic characteristics related to its biological behavior [14–17].

It has been observed in several experiences that radiomic features extracted from MR images have the potential to predict staging, histology, node status, relapse and survival [18–20].

Great interest has been recently paid to radiomics applications focused on CC, with studies predicting treatment response, or long-term outcomes [21–24].

A recent study investigated the potential role of radiomics in predicting pathological complete response after NACRT in LACC patients, reporting promising performances in terms of receiver operating characteristic curve [25].

Prediction of outcome besides treatment response can be useful to tailoring therapeutic treatment scheduling, potentially reducing treatment toxicity in gynecology cancer [26].

Aim of this study is to determine if radiomics features extracted from T2-weighted 1.5 T MRI could predict 2-year local control (2yLC), distant metastasis-free survival (2yDMFS) and overall survival (2yOS) in patients affected by LACC and treated with NACRT followed by radical hysterectomy.

## Materials and methods

### Patient enrollment and treatment

We retrospectively enrolled patients affected by LACC, staged IB2 to IVA from International Federation of Gynecology and Obstetrics (FIGO) 2018, treated with NACRT followed by radical hysterectomy plus pelvic lymphadenectomy after 6–8 weeks.

Patients were recruited from two different institutions: Institution A, an academic tertiary hospital and Institution B, a non-academic tertiary center.

This study was approved by the ethics committee of Institution A.

Inclusion criteria were as follows: histological confirmed invasive carcinoma of the cervix, FIGO stage from IB2 to IVA and absence of distant metastasis.

Patients with incomplete documentation, younger than 18 years, treated with palliative intent and did not undergo surgery were excluded.

**Fig. 1:**
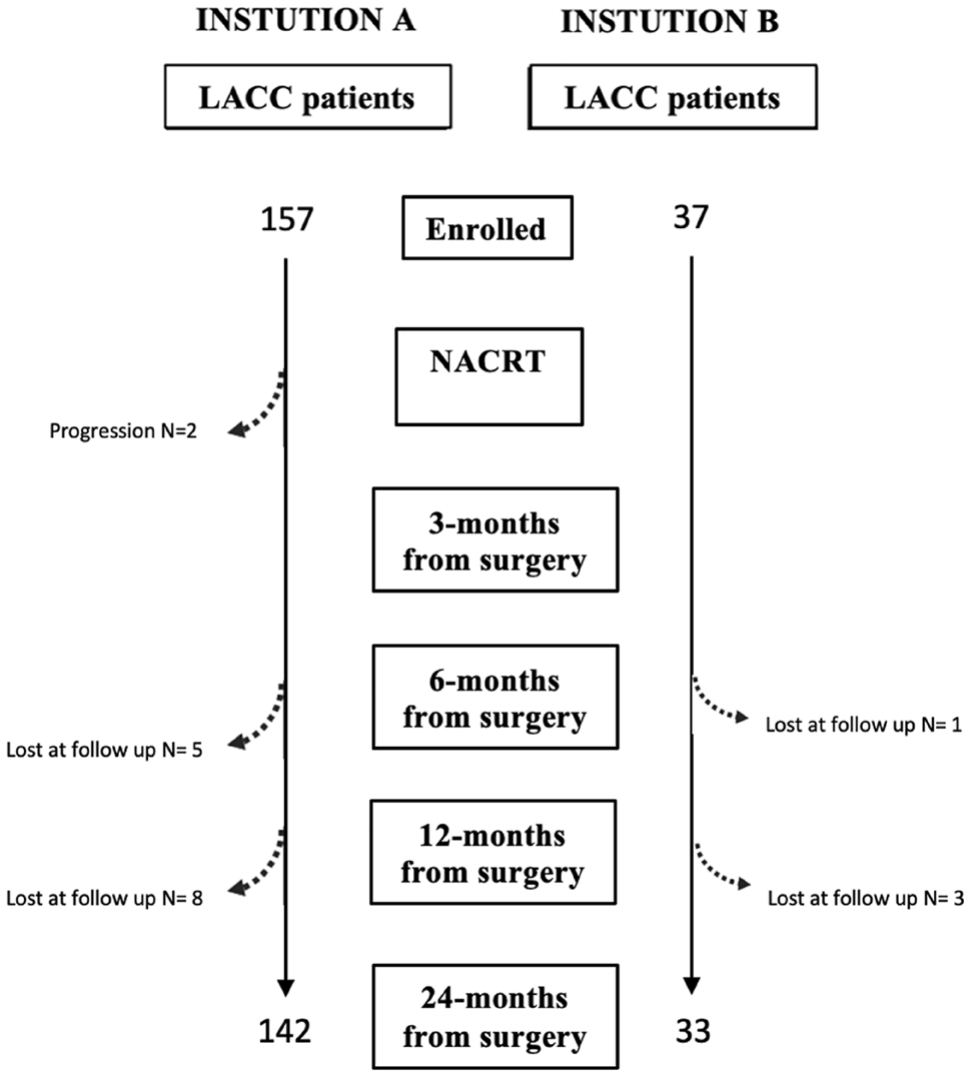
Flowchart of our population

Final cohort of institution A (training set) consisted of 142 patients (from a total of 157, 2 patients have been excluded for progression during NACRT, 13 patients were lost to follow-up); final cohort of institution B (validation set) included 33 patients (from a total of 37 patients, 4 were lost to follow-up) (Fig. 1).

Before treatment, all patients underwent pelvic MRI and total body contrasted enhanced CT scan as staging imaging. 18F-FDG PET-CT was considered and performed only in few selected cases.

All patients included in the analysis underwent NACRT with concurrent weekly cisplatin (Cisplatin dose: 40 mg/sm) alone or plus 5-fluorouracil (Cisplatin dose: 20 mg/sm+5-Fluorouracil dose: 1000 mg) during the first and the last week of treatment.

Radiotherapy volumes were delineated according to consensus guidelines for the clinical target volume (CTV), and organs-at-risk.

CTV was defined as primary tumor (CTV1) plus whole pelvis (CTV2).

In the case of common iliac or lumbo-aortic lymph nodes involvement (cN+) at the staging imaging, lumbo-aortic lymph nodes were also added to the CTV2.

Planning target volumes (PTV) were considered as isotropic expansion of 5 mm from the relative CTV.

Radiotherapy treatment was administered using a simultaneous integrated boost technique delivered in 22 fractions and prescribing 50.6 Gy to PTV1 and 39.6 Gy to PTV2.

All patients were re-evaluated with a pelvic MRI and 18F-FDG PET-CT, according to clinical FIGO stage, for local restaging and subsequently referred to surgery, which was carried out with a laparotomy approach between 7 and 8 weeks from term of radiotherapy.

All patients underwent a Radical hysterectomy ± bilateral annessiectomy performed according to Querleu Morrow’s criteria in relation to the response to radiation treatment, verified by radiological imaging [27].

Systematic pelvic lymphadenectomy was always performed; para-aortic lymph node dissection was added if pelvic or para-aortic lymph node were positive at the pre-treatment imaging or at the intra-operatory frozen section of the pelvic lymph nodes.

Pathological response to treatment was evaluated on surgical specimens.

Pathological complete response was defined as absence of any residual tumor after treatment at any site; microscopic response (pR1) as persistent tumor foci of maximum dimension inferior to 3 mm; macroscopic response (pR2) as persistent tumor foci with maximum dimension exceeding 3 mm [28].

Collected data included age, histology, FIGO stage, presence of positive lymph nodes on PET/CT and pelvic MRI and clinical status at 2 years from surgery.

### Treatment outcome evaluation

Post-treatment surveillance consisted of follow-up visits every 3 months for the first 2 years and every 6 months thereafter.

All the patients with an observation period equal to or longer than 2 years with information relative to diagnostic imaging, surgery and pathological staging were included in the analysis.

Pelvic examination, serum tumor markers, abdominopelvic CT or pelvic MRI or PET-CT scans were performed at each follow-up visit, according to clinical stage.

2yLC, 2yDMFS and 2yOS were considered as treatment outcome, using the end of radiotherapy as reference time point.

Local control outcome (LC) was defined as the interval between the date of treatment completion and the date of tumor local recurrence. Distant metastasis-free survival was defined as the interval between the date of treatment completion and the date of onset of distance recurrence.

Overall survival (OS) was defined as the interval between the date of treatment and the date of cancer related death.

### Image acquisition protocol, image analysis and features extraction

Radiomic analysis was focused on pre-treatment axial T2-weighted MR images, acquired for tumor staging according to institutional staging protocols. The acquisition parameters adopted are reported in Table 1. A total of 564 radiomic features were extracted for each considered clinical outcome.

**Table 1:** Magnetic resonance imaging acquisition parameters used in the MR clinical protocol adopted for axial (AX) acquisitions

	AX T2-W
Sequence	FRFSE
Echo time (ms)	85
NEX	2
Repetition time (ms), TR	4500
No. of sections	30
Receiver bandwidth (kHz)	31.25
Echo train length	26
Field of view (mm), FOV	24
Section thickness (mm)	4
Section spacing (mm)	0.5
Matrix size	384 × 256
b Value (s/mm2)	–
Phase direction	A/P

Gross tumor volume was considered as region of interest; it was manually contoured in consensus by one radiation oncologist and one radiologist, experts in gynecological imaging, using a radiotherapy treatment planning system (Eclipse, Varian Medical Systems, Palo Alto, CA, USA). (

MR images were resampled to a planar resolution of 0.548 × 0.548 mm^2^ and preprocessed using the Laplacian of Gaussian filter, considering the filter widths (σ) ranging from 0 to 4.2 mm with steps of 0.35 mm 4–5. Radiomic analysis was performed using Moddicom, a radiomic software included in the IBSI initiative [19, 29, 30].

Three radiomic features families were extracted for the analysis: morphological features were extracted from raw images, while statistical and textural features were extracted from the filtered one [31, 32].

With regards to the textural features, three gray level matrices were considered: run length (rlm), co-occurrence (cm) and size zone (szm) matrices. The complete list of the extracted radiomic features is reported in the supplementary materials and in similar experiences of our group about this topic [32, 33].

### Statistical analysis

The heterogeneity between training and validation cohorts was evaluated using χ^2^ test for categorical features and t test for continuous ones [31].

A comprehensive database including radiomic features, clinical data and outcomes was created.

For each radiomic feature object of the analysis, the normality of data distribution was evaluated using the Shapiro–Wilk test. The ability of each single feature in predicting at the univariate analysis the dichotomic outcomes object of this study (presence or absence of 2yLC, 2yDFS, 2yOS) was evaluated using the Wilcoxon–Mann–Whitney (WMW) test for features showing data characterized by non-Gaussian distribution and t test for data with Gaussian distribution [34, 35].

Feature showing the lowest *p* value was considered as the most significant feature and selected as first feature for model creation: the use of WMW test was recently considered the most accurate and robust method for feature selection in Radiomics [36]

For each clinical outcome object of this study, different linear logistic regression models with two variables were elaborated, combining the most significant feature at the univariate analysis (lowest *p* value) with all the others radiomic features object of this study [34].

The predictive performance of the different models created was quantified using the area under the receiver operating characteristic (ROC) curve (AUC) [37].

The logistic model showing the highest AUC value was considered as the best predictive model and evaluated on the validation set. The 95% confidence intervals of the AUC value were calculated using the bootstrap method with 2000 iterations.

The best cut-off threshold was identified maximizing the Youden Index (J), and values of sensitivity and specificity at the best threshold were calculated [33, 38].

The statistical analysis was performed using R software (version 3.6.1, Wien Austria) and dedicated packages [39].

## Results

### Clinical characteristics

A total of 175 patients with LACC were analyzed, 142 in the training cohort and 33 in the validation one).

Table 2 summarizes the clinical characteristics of both cohorts.

**Table 2:** Patient characteristic and p value of difference calculated considering t test for continuous variables and chi-square for categorical ones

	Institution A	Institution B	*p* value
	142 pts	33 pts	
Age (mean)	23–76 (51)	28–79 (53)	0.33
*Histology*			
Squamous cell carcinoma	131 (92%)	29(88%)	0.42
Glassy cell squamous carcinoma	0 (%)	1 (3%)	
Clear cell adeno-squamous carcinoma	1 (1%)	0 (0%)	
Adenocarcinoma	10 (7%)	2 (6%)	
Adeno-squamous	0 (%)	1 (3%)	
*FIGO stage*			
IB2	5 (3%)	3 (9%)	0.47
IIA	7 (5%)	2 (6%)	
IIB	116 (82%)	25 (76%)	
IIIA	4 (3%)	2 (6%)	
IIIB	10 (7%)	0 (0%)	
IVA	0 (%)	1 (3%)	
*Nodal status*			
N0	68 (48%)	19 (58%)	0.32
N1	74 (52%)	14 (42%)	
*Pathological response*			
pR0	63 (44%)	11 (33%)	0.32
pR1	41 (29%)	9 (27%)	
pR2	38 (27%)	13 (40%)	

At the end of the follow-up period, 2yLC was observed in 82% of patients in the training cohort and in 78% of patients in the validation cohort; 2yDMFS was observed in 73% of patients in the training cohort and in 82% of patients in the validation cohort; and 2yOS was observed in 86% of patients in the training cohort and in 88% of patients in the validation one.

### Radiomic models

A total of 10 features showed significance (*p* < 0.05) at the univariate analysis for the prediction of 2yOS, 19 features for the 2yLC and 62 for the 2yDMFS. The list of the significant features is reported in the supplementary materials for each outcome.

The two-variables predictive model for 2yDMFS showed moderate performance in training set (0.68) and limited performance in the validation set (0.55).

The predictive model for 2yLC showed moderate performance in both dataset (0.71 in training and validation). The only model showing good performance both in training and validation set was the one predicting 2yOS, with an AUC of 0.77 (95% Confidence interval of 0.70–0.91) in the training set and 0.91 (0.70–1) in the validation set.

The 2yOS model combined two textural features: the correlation based on gray level co-occurrence matrix, calculated after the application of the LoG filter at 0.7 mm and the variance calculated on the size zone matrix after the LOG application at 0.5 mm.

**Fig. 2:**
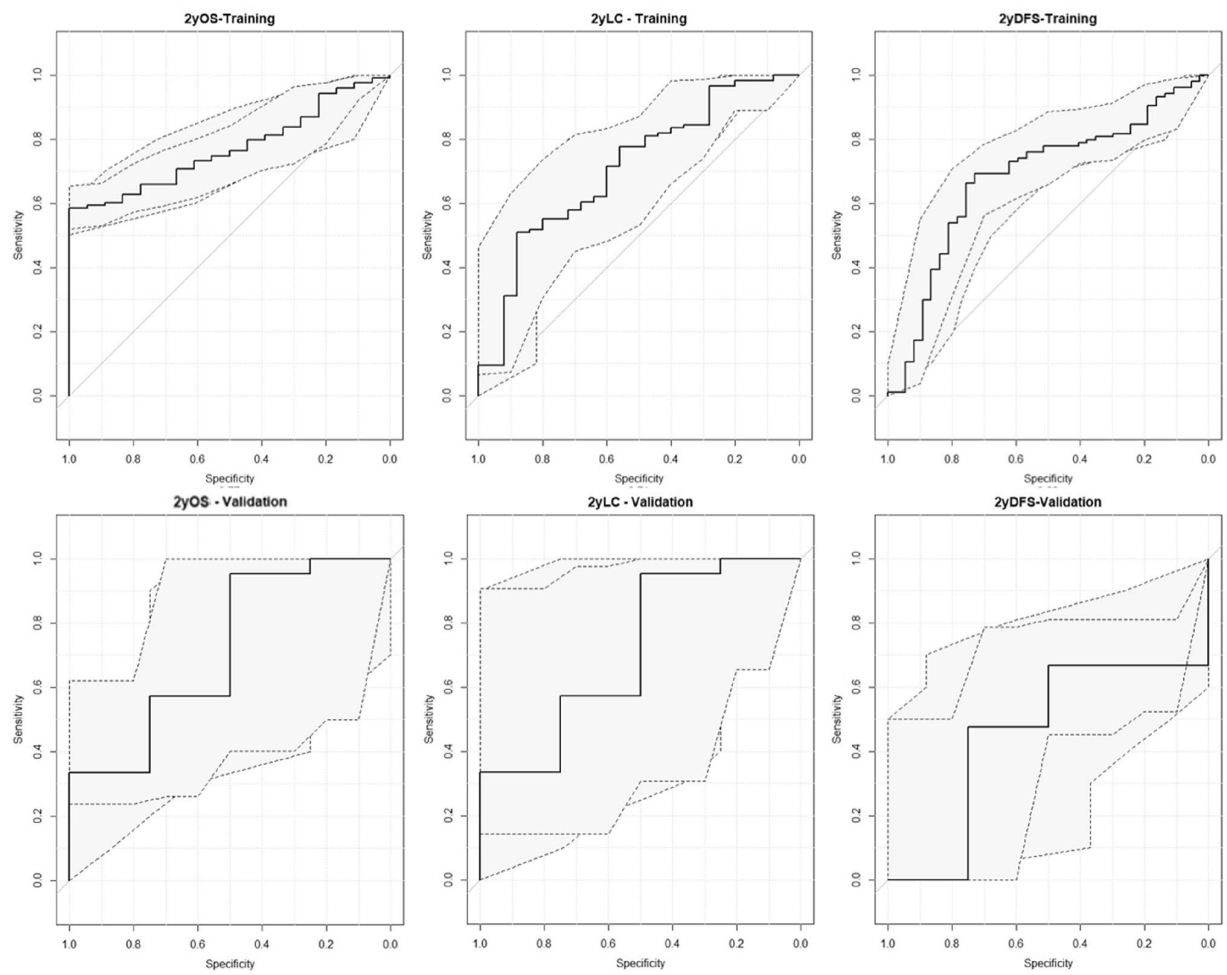
Receiver operating characteristic curve for 2-year overall survival, 2-year distant Metastasis-free survival and 2-year local control in the training set and in the validation set, respectively

The ROC curves of the three models elaborated are reported in Fig. 2, while the predictive performance is summarized in Table 3. The parameters and the coefficient of all the three models are reported in supplementary materials.

**Table 3:** Predictive performance parameters for the three models elaborated in the study

Predictive performance	2yOS		2yDFS		2yLC	
	Training	Validation	Training	Validation	Training	Validation
Sensitivity	58.5	82.6	69.2	47.6	50.9	95.2
Specificity	100.0	100.0	73.0	75.0	88.0	50.0
Threshold	0.9	0.8	0.7	0.8	0.9	0.6
J_index	0.6	0.8	0.4	0.2	0.4	0.5
AUC	77.0	91.3	68.3	55.0	70.9	71.4
Lowest_AUC (95% CI)	0.7	0.7	0.6	0.1	0.6	0.4
Highest_AUC (95% CI)	0.9	1.0	0.8	0.8	0.8	1.0

## Discussion

Several studies aimed to predict and monitor treatment response and clinical outcome analyzing functional imaging, such as MRI or 18-FDG PET-TC in patients affected by cervical cancer [24, 40–42].

In particular, some experiences analyzed temporal variations in tumor heterogeneity patterns on functional imaging such as dynamic contrast enhanced MRI, DWI [24] and FDG PET-CT [42] performed before, during and after CRT course to correlate imaging biomarkers with treatment response outcomes, so defining novel promising prognostic factors.

Our previous experience demonstrated the possibility of using radiomics based on staging MRI to predict pathological complete response after NACRT in cervical cancer. [25]

Despite the important efforts made in terms of treatment response prediction, only few experiences explored the correlation between radiomic predictors and long-terms outcomes, such as distant recurrence or overall survival in cervical cancer [22, 43, 44].

In a study proposed by Ho et al., the mean ADC value extracted from the primary tumor in pre-treatment DWI MR was indicated as a predictor of disease-free survival for CC patients treated with CRT. The authors found that a higher mean ADC value may result in a higher probability of disease-free survival and OS.

The mean ADC value of the primary tumor on pre-treatment MRI was the only imaging feature which resulted to be an independent predictor of disease-free survival [22]

Similar experiences were reported in rectal cancer, where delta radiomic models analyzed the variation of radiomics features extracted from staging and post-treatment MRI to predict tumor behavior and long-term outcomes, such as distant metastasis-free survival and OS [45].

In this study, we aimed to identify radiomic features from staging T2-w MR images which are able to predict 2-year clinical dichotomic outcomes, obtaining promising results in case of OS prediction, but only limited results for LC and distant metastasis--free survival.

In particular, it emerged that the features with the highest performance in predicting the presence of OS after 2 years from the end of treatment are based on the textural analysis.

Being able to predict a long-term outcome from pre-treatment MR images could allow patients to be stratified in different risk groups besides the ones generally used in clinical practice to define the most appropriate treatment schedule (e.g., disease stage).

Furthermore, identifying patients with higher risk of local or distant recurrences could guide clinicians to personalize follow-up schedules or early intercept local or distant recurrence that could represent therapeutic failure. On the other side, less invasive tailored therapeutic strategies may be pursued in patients predicted with low OS probability.

Different limitations burden this work. First of all, the limited replicability of patients setting, as neoadjuvant setting, does not represent the standard of treatment in LACC patient and is not adopted by the largest majority of the centers.

Despite the sound methodology applied (TRIPOD type 3) [46, 47], promising specificity and sensitivity values were reached only in the prediction of 2yOS, this being partly related to the relatively small sample size.

Lastly, the lack of a biological interpretation of the significant features limits the translational value of this experience, even if this issue is not considered mandatory for radiomics study [48].

Future studies are being planned to integrate such evidence with clinical, histopathological and molecular data, which would allow to build multi-omics predictive models to be tested in larger cohorts of patients.

## Conclusion

In this study, a radiomic model was proposed able to predict 2yOS in LACC patients before undergoing NACRT.

An accurate outcome prediction before or during oncological treatments could be an added clinical value to provide a guidance for clinicians in their decision-making process to adapt and tailoring treatment.

To confirm the reliability of these results and translate the use of this model into clinical practice, larger studies with external validation are required.

## Supplementary Information

Below is the link to the electronic supplementary material.

Supplementary file1 (DOCX 35 KB)

## Data Availability

Data will be made available on reasonable request due to restrictions, e.g., privacy or ethical.

## Abbreviations

ADC: Apparent diffusion coefficient
CC: Cervical cancer
CRT: Chemoradiotherapy
DWI: Diffusion-weighted imaging
FIGO: International federation of gynecology and obstetrics
LACC: Locally advanced cervical cancer
MRI: Magnetic resonance imaging
MR: Magnetic resonance
NACRT: Neoadjuvant chemoradiotherapy
OS: Overall survival
PTV: Planned target volume
2yDMFS: Two-year distant metastasis-free survival
2yLC: Two-year local control
2yOS: Two-year overall survival

## References

[1] Sung H, Ferlay J, Siegel RL, Laversanne M, Soerjomataram I, Jemal A (2020). Global cancer statistics 2020: GLOBOCAN estimates of incidence and mortality worldwide for 36 cancers in 185 countries. CA Canc J Clini.

[2] Pötter R, Tanderup K, Kirisits C, de Leeuw A, Kirchheiner K, Nout R (2018). The EMBRACE II study: the outcome and prospect of two decades of evolution within the GEC-ESTRO GYN working group and the EMBRACE studies. Clin Transl Radiat Oncol.

[3] Abu-Rustum NR, Yashar CM, Bean S, Bradley K, Campos SM, Chon HS (2020). NCCN guidelines insights: cervical cancer, version 1. J Nat Compreh Cancer Network JNCCN.

[4] Marth C, Landoni F, Mahner S, McCormack M, Gonzalez-Martin A, Colombo N (2017). Cervical cancer: ESMO clinical practice guidelines for diagnosis, treatment and follow-up. Annal Oncol.

[5] Resbeut M, Cowen D, Viens P, Noirclerc M, Perez T, Gouvernet J (1994). Concomitant chemoradiation prior to surgery in the treatment of advanced cervical carcinoma. Gynecol Oncol.

[6] Colombo PE, Bertrand MM, Gutowski M, Mourregot A, Fabbro M, Saint-Aubert B (2009). Total laparoscopic radical hysterectomy for locally advanced cervical carcinoma (stages IIB, IIA and bulky stages IB) after concurrent chemoradiation therapy: Surgical morbidity and oncological results. Gynecol Oncol.

[7] Ferrandina G, Legge F, Fagotti A, Fanfani F, Distefano M, Morganti A (2007). Preoperative concomitant chemoradiotherapy in locally advanced cervical cancer: Safety, outcome, and prognostic measures. Gynecol Oncol.

[8] Fanfani F, Vizza E, Landoni F, de Iaco P, Ferrandina G, Corrado G (2016). Radical hysterectomy after chemoradiation in FIGO stage III cervical cancer patients versus chemoradiation and brachytherapy: complications and 3-years survival. Eur J Surg Oncol.

[9] Mariagrazia D, Anna F, Gabriella F, Francesco F, Daniela S, Giuseppe D (2005). Preoperative chemoradiotherapy in locally advanced cervical cancer: Long-term outcome and complications. Gynecol Oncol.

[10] Ferrandina G, Gambacorta A, Gallotta V, Smaniotto D, Fagotti A, Tagliaferri L (2014). Chemoradiation With concomitant boosts followed by radical surgery in locally advanced cervical cancer: long-term results of the ROMA-2 prospective phase 2 study. Int J Radiat Oncol Biol Phys.

[11] Sala E, Rockall AG, Freeman SJ, Mitchell DG, Reinhold C (2013). The added role of MR imaging in treatment stratification of patients with gynecologic malignancies: what the radiologist needs to know. Radiology.

[12] Balcacer P, Shergill A, Litkouhi B (2019). MRI of cervical cancer with a surgical perspective: staging, prognostic implications and pitfalls. Abdom Radiol (New York).

[13] Russo L, Gui B, Miccò M, Panico C, De Vincenzo R, Fanfani F (2021). Diagnostic imaging in oncology The role of MRI in cervical cancer > 2 cm (FIGO stage IB2-IIA1) conservatively treated with neoadjuvant chemotherapy followed by conization: a pilot study. Radiol med.

[14] Lambin P, Rios-Velazquez E, Leijenaar R, Carvalho S, Van Stiphout RGPM, Granton P (2012). Radiomics: extracting more information from medical images using advanced feature analysis. Eur J Cancer.

[15] Gillies RJ, Kinahan PE, Hricak H (2016). Radiomics: images are more than pictures, they are data. Radiology.

[16] Lambin P, Leijenaar RTH, Deist TM, Peerlings J, de Jong EEC, van Timmeren J (2017). Radiomics: the bridge between medical imaging and personalized medicine. Nat Rev Clin Oncol.

[17] Liu Z, Wang S, Dong D, Wei J, Fang C, Zhou X (2019). The applications of radiomics in precision diagnosis and treatment of oncology: opportunities and challenges. Theranostics.

[18] Nardone V, Boldrini L, Grassi R, Franceschini D, Morelli I, Becherini C (2021). Radiomics in the setting of neoadjuvant radiotherapy: a new approach for tailored treatment. Cancers.

[19] Cusumano D, Dinapoli N, Boldrini L, Chiloiro G, Gatta R, Masciocchi C (2018). Fractal-based radiomic approach to predict complete pathological response after chemo-radiotherapy in rectal cancer. Radiologia Medica.

[20] Boldrini L, Cusumano D, Chiloiro G, Casà C, Masciocchi C, Lenkowicz J (2019). Delta radiomics for rectal cancer response prediction with hybrid 0.35 T magnetic resonance-guided radiotherapy (MRgRT): a hypothesis-generating study for an innovative personalized medicine approach. La Radiologia Medica.

[21] Fang M, Kan Y, Dong D, Yu T, Zhao N, Jiang W (2020). Multi-habitat based radiomics for the prediction of treatment response to concurrent chemotherapy and radiation therapy in locally advanced cervical cancer. Front Oncol.

[22] Ho JC, Allen PK, Bhosale PR, Rauch GM, Fuller CD, Mohamed ASR (2017). Diffusion-weighted MRI as a predictor of outcome in cervical cancer following chemoradiation. Int J Radiat Oncol Biol Phys.

[23] Perrone AM, Dondi G, Coe M, Ferioli M, Telo S, Galuppi A (2020). Predictive role of MRI and 18F FDG PET response to concurrent chemoradiation in T2b cervical cancer on clinical outcome: a retrospective single center study. Cancers.

[24] Bowen SR, Yuh WTC, Hippe DS, Wu W, Partridge SC, Elias S (2018). Tumor radiomic heterogeneity: multiparametric functional imaging to characterize variability and predict response following cervical cancer radiation therapy. J Magn Reson Imaging.

[25] Gui B, Autorino R, Miccò M, Nardangeli A, Pesce A, Lenkowicz J (2021). Pretreatment MRI radiomics based response prediction model in locally advanced cervical cancer. Diagnostics.

[26] Albano D, Benenati M, Bruno A, Bruno F, Calandri M, Caruso D (2021). Imaging side effects and complications of chemotherapy and radiation therapy: a pictorial review from head to toe. Insight Imag.

[27] Querleu D, Morrow CP (2008). Classification of radical hysterectomy. Lancet Oncol.

[28] Zannoni GF, Vellone VG, Carbone A (2008). Morphological effects of radiochemotherapy on cervical carcinoma: a morphological study of 50 cases of hysterectomy specimens after neoadjuvant treatment. Int J Gynecol Pathol: Offi J Int Soci Gynecol Pathol.

[29] Gatta R, Vallati M, Dinapoli N, Masciocchi C, Lenkowicz J, Cusumano D (2019). Towards a modular decision support system for radiomics: a case study on rectal cancer. Artif Intell Med.

[30] Cusumano D, Meijer G, Lenkowicz J, Chiloiro G, Boldrini L, Masciocchi C (2021). A field strength independent MR radiomics model to predict pathological complete response in locally advanced rectal cancer. Radiol Medica.

[31] Zwanenburg A, Vallières M, Abdalah MA, Aerts HJWL, Andrearczyk V, Apte A (2020). The image biomarker standardization initiative: standardized quantitative radiomics for high-throughput image-based phenotyping. Radiology.

[32] Cusumano D, Boldrini L, Yadav P, Yu G, Musurunu B, Chiloiro G (2021). Delta radiomics for rectal cancer response prediction using low field magnetic resonance guided radiotherapy: an external validation. Physica Med.

[33] Cusumano D, Boldrini L, Yadav P, Yu G, Musurunu B, Chiloiro G (2020). External validation of early regression index (ERITCP) as predictor of pathologic complete response in rectal cancer using magnetic resonance-guided radiation therapy. Int J Radiat Oncol Biol Phys.

[34] Taylor J (1997). Introduction to error analysis, the study of uncertainties in physical measurements.

[35] Cusumano D, Boldrini L, Yadav P, Casà C, Lee SL, Romano A (2021). Delta radiomics analysis for local control prediction in pancreatic cancer patients treated using magnetic resonance guided radiotherapy. Diagnostics.

[36] Parmar C, Grossmann P, Bussink J, Lambin P, Aerts HJWL (2015). Machine learning methods for quantitative radiomic biomarkers. Sci Rep.

[37] ICRU Report 79, Receiver Operating Characteristic (ROC) Analysis in Medical Imaging – ICRU n.d. https://www.icru.org/report/receiver-operating-characteristic-roc-analysis-in-medical-imaging-icru-report-79/ (accessed December 1, 2021)

[38] Ruopp MD, Perkins NJ, Whitcomb BW, Schisterman EF (2008). Youden Index and optimal cut-point estimated from observations affected by a lower limit of detection. Biometr J Biometr Zeitsch.

[39] Robin X, Turck N, Hainard A, Tiberti N, Lisacek F, Sanchez JC (2011). pROC: an open-source package for R and S+ to analyze and compare ROC curves. BMC Bioinfor.

[40] Schwarz JK, Siegel BA, Dehdashti F, Grigsby PW (2012). Metabolic response on post-therapy FDG-PET predicts patterns of failure after radiotherapy for cervical cancer. Int J Radiat Oncol Biol Phys.

[41] Lima GM, Matti A, Vara G, Dondi G, Naselli N, De Crescenzo EM (2018). Prognostic value of posttreatment 18F-FDG PET/CT and predictors of metabolic response to therapy in patients with locally advanced cervical cancer treated with concomitant chemoradiation therapy: an analysis of intensity- and volume-based PET parameters. Eur J Nucl Med Mol Imaging.

[42] Yang F, Thomas MA, Dehdashti F, Grigsby PW (2013). Temporal analysis of intratumoral metabolic heterogeneity characterized by textural features in cervical cancer. Eur J Nucl Med Molecul Imag.

[43] Lucia F, Visvikis D, Desseroit MC, Miranda O, Malhaire JP, Robin P (2018). Prediction of outcome using pretreatment 18F-FDG PET/CT and MRI radiomics in locally advanced cervical cancer treated with chemoradiotherapy. Eur J Nucl Med Mol Imag.

[44] Yao A, Haiyan Z, Congying X, Xiance J (2020). Radiomics in cervical cancer: Current applications and future potential. Critical Rev OncolHematol.

[45] Chiloiro G, Rodriguez-Carnero P, Lenkowicz J, Casà C, Masciocchi C, Boldrini L (2020). Delta radiomics can predict distant metastasis in locally advanced rectal cancer: the challenge to personalize the cure. Front Oncol.

[46] Collins GS, Reitsma JB, Altman DG, Moons KGM (2015). Transparent reporting of a multivariable prediction model for individual prognosis or diagnosis (TRIPOD): the TRIPOD statement. Ann Intern Med.

[47] Localio AR, Stack CB (2015). TRIPOD: a new reporting baseline for developing and interpreting prediction models. Ann Inter Med.

[48] Fournier L, Costaridou L, Bidaut L, Michoux N, Lecouvet FE, de Geus-Oei LF (2021). Incorporating radiomics into clinical trials: expert consensus endorsed by the european society of radiology on considerations for data-driven compared to biologically driven quantitative biomarkers. Eur Radiol.

