# Supplementary material for: Radiomics-based prediction of two-year clinical outcome in locally advanced cervical cancer patients undergoing neoadjuvant chemoradiotherapy
Source: Radiol Med. 2022 Mar 24;127(5):498–506. doi: 10.1007/s11547-022-01482-9 (PMC9098600; doi:10.1007/s11547-022-01482-9)
Supplement: Supplementary file 1 — Supplementary file1 (DOCX 35 KB) [file 11547_2022_1482_MOESM1_ESM.docx]

**Supplementary materials**

The complete list of the 92 features extracted is reported below, divided by family. The id reported in the list is the same reported in the biomarker initiative refence manual, where it is possible to found the mathematical formulation of each feature [36].

- **Morphological-based**
  - 4.1.1 Volume
  - 4.1.3 Surface area
  - 4.1.4 Surface to volume ratio
  - 4.1.5 Compactness 1
  - 4.1.6 Compactness 2
  - 4.1.7 Spherical disproportion
  - 4.1.8 Sphericity
  - 4.1.9 Asphericity
  - 4.1.10 Centre of mass shift
  - 4.1.11 Maximum 3D diameter
  - 4.1.12 Major axis length
  - 4.1.13 Minor axis length
  - 4.1.14 Least axis length
- **Intensity-based**
  - 4.1.15 Elongation
  - 4.1.16 Flatness
  - 4.3.1 Mean
  - 4.3.2 Variance
  - 4.3.3 Skewness
  - 4.3.4 Kurtosis
  - 4.3.5 Median
  - 4.3.6 Minimum grey level
  - 4.3.7 10th percentile
  - 4
  - .3.8 90th percentile
  - 4.3.9 Maximum grey level
  - 4.3.10 Interquartile range
  - 4.3.11 Range
  - 4.3.12 Mean absolute deviation
  - 4.3.13 Robust mean absolute deviation
  - 4.3.17 Energy
  - 4.3.18 Root mean square
  - 4.4.18 Entropy
  - 4.4.19 Uniformity
- **Grey level co-occurrence based features–Texture features (GLCM)**
  - 4.6.1 Joint maximum
  - 4.6.2 Joint average
  - 4.6.3 Joint variance
  - 4.6.4 Joint entropy
  - 4.6.5 Difference average
  - 4.6.6 Difference variance
  - 4.6.7 Difference entropy
  - 4.6.8 Sum average
  - 4.6.9 Sum variance
  - 4.6.10 Sum entropy
  - 4.6.11 Angular second moment
  - 4.6.12 Contrast
  - 4.6.13 Dissimilarity
  - 4.6.14 Inverse difference
  - 4.6.15 Inverse difference normalised
  - 4.6.16 Inverse difference moment
  - 4.6.17 Inverse difference moment normalised
  - 4.6.18 Inverse variance
  - 4.6.19 Correlation
  - 4.6.20 Autocorrelation
  - 4.6.21 Cluster tendency
  - 4.6.22 Cluster shade
  - 4.6.23 Cluster prominence
  - 4.6.24 First measure of information correlation
  - 4.6.25 Second measure of information correlation
- **Grey level run length based features–Texture features (GLRLM)**
  - 4.7.1 Short runs emphasis
  - 4.7.2 Long runs emphasis
  - 4.7.3 Low grey level run emphasis
  - 4.7.4 High grey level run emphasis
  - 4.7.5 Short run low grey level emphasis
  - 4.7.6 Short run high grey level emphasis
  - 4.7.7 Long run low grey level emphasis
  - 4.7.8 Long run high grey level emphasis
  - 4.7.9 Grey level non-uniformity
  - 4.7.10 Grey level non-uniformity normalised
  - 4.7.11 Run length non-uniformity
  - 4.7.12 Run length non-uniformity normalised
  - 4.7.13 Run percentage
  - 4.7.14 Grey level variance
  - 4.7.15 Run length variance
  - 4.7.16 Run entropy
- **Grey level size zone based features–Texture features (GLDZM)**
  - 4.8.1 Small zone emphasis
  - 4.8.2 Large zone emphasis
  - 4.8.3 Low grey level zone emphasis
  - 4.8.4 High grey level zone emphasis
  - 4.8.5 Small zone low grey level emphasis
  - 4.8.6 Small zone high grey level emphasis
  - 4.8.7 Large zone low grey level emphasis
  - 4.8.8 Large zone high grey level emphasis
  - 4.8.9 Grey level non-uniformity
  - 4.8.10 Grey level non-uniformity normalised
  - 4.8.11 Zone size non-uniformity
  - 4.8.12 Zone size non-uniformity normalised
  - 4.8.13 Zone percentage
  - 4.8.14 Grey level variance
  - 4.8.15 Zone size variance
  - 4.8.16 Zone size entropy

**Overall Survival after 2 years**

*Significant features*

| **Feature Family** | **Feature Name** | **Log Filter** | **p-value** |
| --- | --- | --- | --- |
| Textural-GLCM | Correlation | 0,7 | 0,001 |
| Textural-GLCM | Correlation | 0,6 | 0,001 |
| Textural-GLCM | Correlation | 0,8 | 0,002 |
| Textural-GLCM | Correlation | 0,9 | 0,003 |
| Textural-GLCM | Correlation | 1 | 0,004 |
| Morphological | Spherical disproportion | None | 0,013 |
| Morphological | Asphericity | None | 0,013 |
| Morphological | Sphericity | None | 0,019 |
| Morphological | Compactness 1 | None | 0,019 |
| Morphological | Compactness 2 | None | 0,022 |

*Summary of the model*

|  | Estimate | Std. | Error | z |
| --- | --- | --- | --- | --- |
| (Intercept) | 22,372 | 6,529 | 3,427 | 0,001 |
| corr_07 | -28,355 | 9,004 | -3,149 | 0,002 |
| zs_var_05 | -1,622 | 0,537 | -3,020 | 0,003 |

*Performance Metrics*

|  | **Sensitivity** | **Specificity** | **Threshold** | **J_index** | **AUC** | **Low_AUC** | **High_AUC** |
| --- | --- | --- | --- | --- | --- | --- | --- |
| Training | 58,5 | 100,0 | 0,9 | 0,6 | 77,0 | 0,7 | 0,9 |
| Validation | 82,6 | 100,0 | 0,8 | 0,8 | 91,3 | 0,7 | 1,0 |

**Disease free survival after 2 years**

*Significant features*

| **Feature Family** | **Feature Name** | **Log Filter** | **p-value** |
| --- | --- | --- | --- |
| Statistical | entropy | 0,9 | 0,004 |
| Statistical | entropy | 1 | 0,004 |
| Textural-GLSZM | lgze | 0,5 | 0,005 |
| Textural-GLSZM | szlge | 0,5 | 0,008 |
| Morphological | Lminor | None | 0,008 |
| Statistical | entropy | 0,8 | 0,008 |
| Morphological | surface | None | 0,010 |
| Textural-GLCM | clust.prom | 0,5 | 0,011 |
| Textural-GLCM | clust.prom | 0,7 | 0,013 |
| Textural-GLCM | diff.var | 0,8 | 0,015 |
| Morphological | Lleast | None | 0,015 |
| Textural-GLCM | diff.var | 0,5 | 0,017 |
| Textural-GLSZM | szlge | 0,6 | 0,017 |
| Textural-RLM | rlnu | None | 0,017 |
| Textural-GLCM | diff.var | 0,6 | 0,017 |
| Statistical | entropy | 0,7 | 0,018 |
| Morphological | volume | None | 0,019 |
| Textural-GLSZM | zsnu.norm | 0,8 | 0,019 |
| Textural-GLCM | diff.var | 0,7 | 0,020 |
| Textural-GLCM | diff.entr | 0,6 | 0,020 |
| Textural-GLCM | diff.var | 0,9 | 0,021 |
| Textural-GLSZM | lgze | 0,6 | 0,022 |
| Morphological | Lmajor | None | 0,022 |
| Textural-GLCM | diff.var | 1 | 0,022 |
| Textural-GLSZM | szlge | 0,7 | 0,022 |
| Textural-GLSZM | gl.var | 0,7 | 0,023 |
| Textural-GLSZM | sze | 0,8 | 0,023 |
| Textural-GLCM | clust.prom | 0,6 | 0,023 |
| Textural-GLSZM | lzlge | 0,5 | 0,024 |
| Textural-GLSZM | sze | 1 | 0,024 |
| Textural-GLCM | joint.var | 0,7 | 0,026 |
| Textural-RLM | gl.var | 0,7 | 0,026 |
| Textural-GLSZM | glnu | None | 0,027 |
| Textural-GLCM | info.corr.1 | 0,6 | 0,027 |
| Textural-RLM | lrlge | 0,5 | 0,027 |
| Textural-GLCM | contrast | 1 | 0,028 |
| Textural-RLM | srlge | 0,5 | 0,028 |
| Textural-RLM | lgre | 0,5 | 0,029 |
| Textural-GLCM | joint.var | 0,8 | 0,030 |
| Statistical | entropy | 0,5 | 0,032 |
| Statistical | uniformity | 0,5 | 0,032 |
| Textural-GLSZM | lgze | 0,7 | 0,034 |
| Textural-GLCM | clust.tend | 0,5 | 0,035 |
| Textural-GLCM | sum.var | 0,5 | 0,035 |
| Textural-GLSZM | gl.var | 0,8 | 0,036 |
| Textural-GLCM | clust.prom | 0,9 | 0,037 |
| Textural-GLSZM | gl.var | 0,5 | 0,037 |
| Textural-GLCM | contrast | 0,5 | 0,038 |
| Textural-RLM | glnu.norm | 0,8 | 0,039 |
| Textural-RLM | rlnu.norm | 1 | 0,039 |
| Textural-GLCM | dissimilarity | 0,8 | 0,040 |
| Textural-GLSZM | lgze | 0,8 | 0,040 |
| Textural-RLM | srlge | 0,7 | 0,041 |
| Textural-GLSZM | zsnu.norm | 1 | 0,041 |
| Textural-GLSZM | zsnu | 0,7 | 0,041 |
| Textural-GLCM | clust.tend | 0,6 | 0,042 |
| Textural-GLCM | sum.var | 0,6 | 0,042 |
| Textural-GLCM | diff.entr | 1 | 0,043 |
| Textural-RLM | srlge | 0,8 | 0,043 |
| Textural-RLM | srlge | 0,9 | 0,043 |
| Textural-RLM | lrlge | 0,5 | 0,045 |
| Textural-GLCM | inv.diff.mom | 0,8 | 0,049 |

*Summary of the model*

|  | Estimate | Std. | Error | z |
| --- | --- | --- | --- | --- |
| (Intercept) | -2,7E+00 | 1,7E+00 | -1,6E+00 | 1,1E-01 |
| Entropy_0,9 | 1,8E+00 | 8,5E-01 | 2,1E+00 | 3,4E-02 |
| Cluster Shade Cross Matrix | 3,3E-05 | 2,9E-05 | 1,1E+00 | 2,6E-01 |

*Performance Metrics*

|  | **Sensitivity** | **Specificity** | **Threshold** | **J_index** | **AUC** | **Low_AUC** | **High_AUC** |
| --- | --- | --- | --- | --- | --- | --- | --- |
| Training | 69,2 | 73,0 | 0,7 | 0,4 | 68,3 | 0,6 | 0,8 |
| Validation | 47,6 | 75,0 | 0,8 | 0,2 | 45,2 | 0,1 | 0,8 |

**Local Control after 2 years**

*Significant features*

| **N** | **Feature Family** | **Feature Name** | **Log Filter** | **p-value** |
| --- | --- | --- | --- | --- |
| 1 | Morphological | Compactness | None | 0,009 |
| 2 | Morphological | Compactness | None | 0,011 |
| 3 | Morphological | Sphericity | None | 0,012 |
| 5 | Morphological | Asphericity | None | 0,014 |
| 4 | Morphological | Sphericity Disproportion | None | 0,014 |
| 6 | Textural-GLCM | Correlation | 1 | 0,017 |
| 7 | Morphological | Surphace | None | 0,020 |
| 8 | Textural-GLCM | Correlation | 0,9 | 0,021 |
| 9 | Textural-GLCM | Variance Difference | 0,8 | 0,023 |
| 10 | Textural-GLCM | Variance Difference | 0,9 | 0,025 |
| 11 | Textural-GLCM | Correlation | 0,8 | 0,026 |
| 12 | Textural-GLCM | Correlation | 0,7 | 0,026 |
| 13 | Textural-GLCM | Variance Difference | 1 | 0,033 |
| 15 | Textural-GLCM | Contrast | 0,9 | 0,034 |
| 14 | Textural-GLCM | Variance Difference | 0,7 | 0,034 |
| 16 | Textural-GLCM | Contrast | 0,8 | 0,035 |
| 17 | Textural-GLCM | Contrast | 1 | 0,041 |
| 18 | Morphological | Minor Length | None | 0,042 |
| 19 | Textural-GLRLM | RLNU | None | 0,044 |

*Summary of the model*

|  | Estimate | Std. | Error | z |
| --- | --- | --- | --- | --- |
| (Intercept) | -2,49432 | 1,41408 | -1,764 | 0,0777 |
| Compactness | 137,12289 | 56,6514 | 2,42 | 0,0155 |
| Variance_1 | 0,0418 | 0,03641 | 1,148 | 0,2509 |

*Performance Metrics*

|  | **Sensitivity** | **Specificity** | **Threshold** | **J_index** | **AUC** | **Low_AUC** | **High_AUC** |
| --- | --- | --- | --- | --- | --- | --- | --- |
| Training | 50,9 | 88,0 | 0,9 | 0,4 | 70,9 | 0,6 | 0,8 |
| Validation | 95,2 | 50,0 | 0,6 | 0,5 | 71,4 | 0,4 | 1,0 |
